# Detecting Smoking Events Using Accelerometer Data Collected Via Smartwatch Technology: Validation Study

**DOI:** 10.2196/mhealth.9035

**Published:** 2017-12-13

**Authors:** Casey A Cole, Dien Anshari, Victoria Lambert, James F Thrasher, Homayoun Valafar

**Affiliations:** ^1^ Computational Biology Research Group Department of Computer Science University of South Carolina Columbia, SC United States; ^2^ Department of Health Promotion, Education & Behavior Arnold School of Public Health University of South Carolina Columbia, SC United States

**Keywords:** machine learning, neural networks, automated pattern recognition, smoking cessation, ecological momentary assessment, digital signal processing, data mining

## Abstract

**Background:**

Smoking is the leading cause of preventable death in the world today. Ecological research on smoking in context currently relies on self-reported smoking behavior. Emerging smartwatch technology may more objectively measure smoking behavior by automatically detecting smoking sessions using robust machine learning models.

**Objective:**

This study aimed to examine the feasibility of detecting smoking behavior using smartwatches. The second aim of this study was to compare the success of observing smoking behavior with smartwatches to that of conventional self-reporting.

**Methods:**

A convenience sample of smokers was recruited for this study. Participants (N=10) recorded 12 hours of accelerometer data using a mobile phone and smartwatch. During these 12 hours, they engaged in various daily activities, including smoking, for which they logged the beginning and end of each smoking session. Raw data were classified as either smoking or nonsmoking using a machine learning model for pattern recognition. The accuracy of the model was evaluated by comparing the output with a detailed description of a modeled smoking session.

**Results:**

In total, 120 hours of data were collected from participants and analyzed. The accuracy of self-reported smoking was approximately 78% (96/123). Our model was successful in detecting 100 of 123 (81%) smoking sessions recorded by participants. After eliminating sessions from the participants that did not adhere to study protocols, the true positive detection rate of the smartwatch based-detection increased to more than 90%. During the 120 hours of combined observation time, only 22 false positive smoking sessions were detected resulting in a 2.8% false positive rate.

**Conclusions:**

Smartwatch technology can provide an accurate, nonintrusive means of monitoring smoking behavior in natural contexts. The use of machine learning algorithms for passively detecting smoking sessions may enrich ecological momentary assessment protocols and cessation intervention studies that often rely on self-reported behaviors and may not allow for targeted data collection and communications around smoking events.

## Introduction

Despite rapid adoption of many tobacco control policies around the world, cigarette smoking remains the greatest preventable cause of death [1]. Ecological momentary assessment studies are increasingly popular for understanding smoking behavior in context [2-4]. Studies in this area have traditionally relied on participants to self-report smoking behaviors in real time, which can be particularly burdensome for heavier smokers and result in missing or biased information if participants are not forthcoming about or forget smoking events [5]. In this study, a smoking event can be either an individual puff or an entire session, defined as the time it takes to smoke a single cigarette. Emerging technologies that allow for passive detection of stereotyped behaviors such as smoking may be able to decrease or eliminate reliance on burdensome and potentially biased self-reports to study when, how frequently, and under what circumstances smoking behavior occurs.

Mobile phones and, recently, smartwatch technologies have rapidly spread and are widely available [6]. Typical smartwatches house sophisticated sensors that accurately track simple activities, such as step counting. In recent years, methods have been developed that use these sensors to detect more complex activities, such as eating and drinking [7,8]. Previous research [9-12] has shown the possibility of detecting smoking using smart devices. However, these studies have employed highly intrusive devices such as respiration bands [10,13] worn across the chest and two-lead electrocardiographs worn under the clothes to achieve high accuracy in detection. In our previous, laboratory-based work [14,15], we have shown that smoking can also be detected by leveraging the accelerometer sensor found on a typical smartwatch in conjunction with common machine learning algorithms.

The utilization of smartwatches presents a nonintrusive means of smoking detection that potentially eliminates the need for reliance on self-reporting. The purpose of this study is to extend our previous laboratory-based work to determine the feasibility and accuracy of our detection method with a population of smokers wearing the device in the natural context of normal daily activities.

## Methods

### Overview

Adult smokers were recruited to wear a commonly available smartwatch while recording their daily activities, including smoking and other behaviors that are similar to smoking (ie, eating, drinking). The data from these recordings were then used in a machine learning exercise to develop an automated gesture detection algorithm. The accuracy of our automated detection was compared against the self-reported information on activities and manual inspection of smoking session data.

### Recruitment of Participants

Participants were recruited through flyers, which included study information and a link to an online eligibility survey that was accessible via a clickable URL address and a QR code. The survey asked about participants’ smoking behavior as well as age, gender, and contact information. Eligibility criteria included age older than 18 years, having smoked at least 100 cigarettes in their life, smoking more than 10 cigarettes daily, and preference for smoking with the right hand. The flyers were posted throughout Columbia, SC, in areas where smokers were likely to congregate (eg, coffee shops, bars), as well as online venues such as Craigslist. The incentive for completion of the study was a US $100 Visa gift card that was given to each participant after concluding the protocol.

Only participants who met all eligibility requirements were contacted and invited to a study briefing. In the briefing, participants’ eligibility was reconfirmed with a smoke carbon monoxide breathalyzer. A level of 8 ppm was used as the cutoff, which is slightly higher than cutoff levels of 5 to 6 ppm suggested for distinguishing smokers from nonsmokers in other studies [16-18]. Participants were provided with an Asus Zenwatch and Android mobile phone to complete the trial. A 15-minute tutorial was given to each participant on how to use the data collection app and smartwatch and how to fill in the smoking logs using their mobile phone to register the times when they began and finished smoking. Fourteen smokers attended a briefing; two did not meet the criteria of 8 ppm after taking the smoke carbon monoxide breathalyzer measure and were excluded from the study. Of these 12 participants, data from two were inconsistent and excluded from the analysis because they did not follow the study procedures. In one case, the participant wore the watch on the left hand instead of the right hand and therefore did not collect data from the hand used to smoke. In the second case, large sections of data were missing due to the participant losing Bluetooth connectivity between their watch and their phone by moving more than 30 feet away from the phone. Hence, data from 10 participants were analyzed.

After the study was completed, these 10 participants were asked to fill out a brief demographic survey. The survey included basic questions about age, race, ethnicity, gender, and intentions to quit or continue smoking.

### Data Collection and Annotation

**Figure 1:**
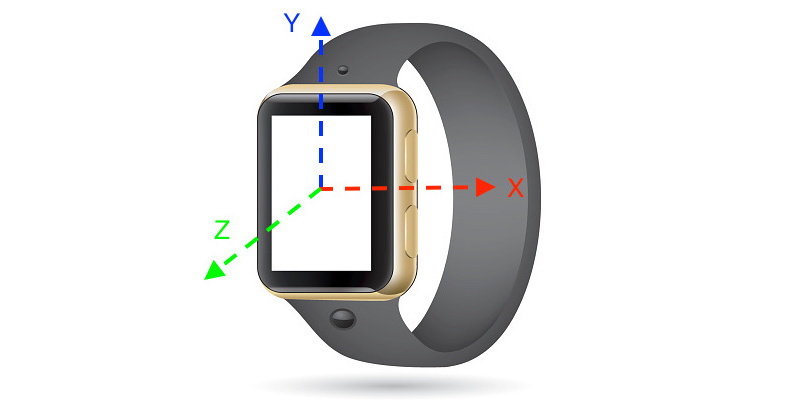
An illustration of accelerometer axes on a typical smartwatch.

The data analyzed in this study consisted of the three-dimensional accelerometer data collected from the Asus Zenwatch (first generation). The accelerometer onboard the Asus Zenwatch is triaxial and therefore capable of recording acceleration in three principal axes x, y, and z. These three axes are situated on the watch as shown in Figure 1, where the z-axis (in green) is perpendicular to the watch face.

Although a few apps exist for recording accelerometer data on both Apple and Android platforms, none of them contained the required features, such as recording and transmission of the data to cloud storage or alteration of sampling frequency. Therefore, we developed an app capable of recording, maintaining, and transmitting data to Dropbox as the means of data collection and storage across our cohort of participants. The use of a customized app allowed for control over the sampling frequency of the data. During this investigation, a fixed sampling frequency of 20 Hz was used.

Each participant was asked to record a total of 12 hours of data over the course of three days. The total of 12 hours was partitioned into seven periods: four 1-hour periods, two 2-hour periods, and one 4-hour period. The participants were instructed to schedule these seven periods such that each would contain at least one full smoking session. Due to the large data transfers occurring between the watch and the phone, the battery life of the watch was not able to achieve the full 4 hours in most cases. In these cases, the participants were asked to record as long as they could until the battery power was nearly depleted.

In addition to the accelerometer data, the participants were instructed to record the beginning and end times of each cigarette in an online logbook using the provided mobile phone. A bookmark on the phones linked to a brief Google form that served as their logbook. The protocol involved recording the starting timestamp immediately before beginning a smoking session. In addition, each participant was asked to indicate whether the cigarette was the first from a new pack. After each smoking session, they were asked to report the end of their smoking session as well as the approximate number of puffs during their smoking session.

Smoking sessions were extracted and inspected based on the start and end times recorded in each participant’s log entries. The duration of these sessions ranged from 2 to 20 minutes in length. However, these ranges are misleading in some ways. For instance, some of the longer sessions (>10 minutes) clearly consisted of more than one smoking event. This behavior is typical for chain-smokers but, as per our defined protocol, should have been recorded as two separate sessions instead of one. Any other gesture that was not within one of the reported sessions was classified as a nonsmoking session.

### A Hierarchical Approach to Detection of the Smoking Gesture

Machine learning techniques have been commonly used in the broad field of pattern recognition. Common machine learning techniques consist of naive Bayes, support vector machine, decision tree, random forest, artificial neural network, and rule-based artificial intelligence (AI), to name a few. In this study, we have integrated artificial neural networks and rule-based AI in a hierarchical fashion to improve recognition of smoking activity.

#### Artificial Neural Networks

**Figure 2:**
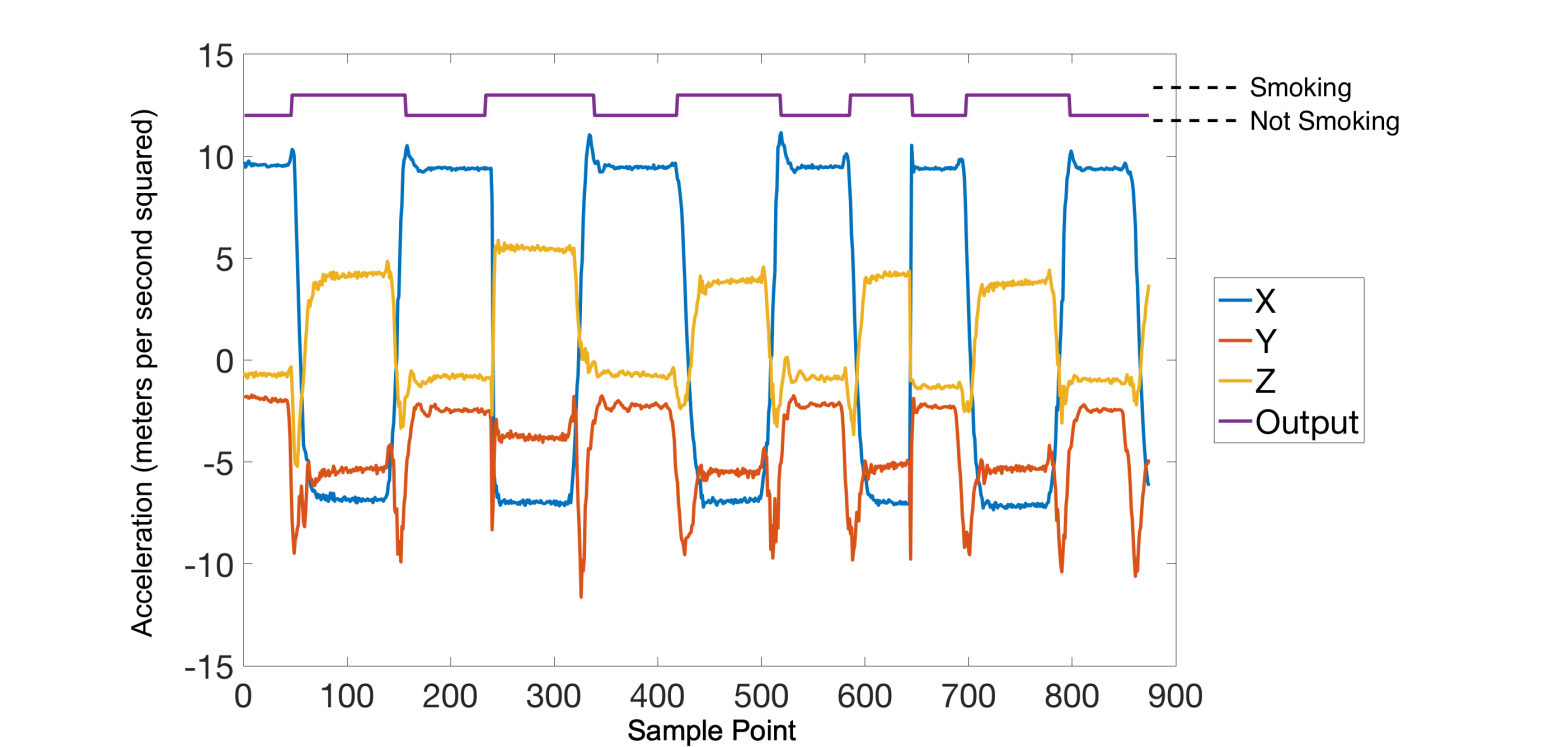
An example of a smoking session. Each dimension of the accelerometer data is shown in blue (x), red (y), and yellow (z). An ideal output of the artificial neural network is shown in purple where each bump denotes a smoking gesture.

In this study, two-layer, feed-forward artificial neural networks [19] with 10 hidden neurons were used as the core engine for detection of smoking gestures. Typically, the creation of an artificial neural network occurs in two main steps: training and validation. Details about the training and validation processes can be found in our previous works [14,15]. In general, the artificial neural network was trained to produce an output of 1 during the smoking gesture and a 0 during all other activities. Figure 2 provides an illustration of a sample smoking session (with five distinct puffs) and the expected ideal output. In this figure, the patterns illustrated in blue, red, and yellow correspond to the x, y, and z dimensions of the accelerometer data and the pattern shown in purple denotes the ideal output.

#### Rule-Based Artificial Intelligence

Rule-based AI constitutes the earliest form of the machine learning techniques. Rule-based techniques can be very efficient in circumstances where the actions taken by the AI core can be deduced based on a set of definable rules. The cooperation between the artificial neural network and rule-based cores can be structured in a variety of ways. In our study, we chose a hierarchical model, where the artificial neural network operates as the core of the smoking detection and rule-based AI operates in a layer above the artificial neural network. In this arrangement, the rule-based core is responsible for establishing the beginning and the end of a “puff” gesture, counting the number of puffs, and establishing the beginning and end of a new smoking session. The rule-based layer also addresses some of the shortcomings of our previous studies [14], where several nonsmoking gestures (eg, scratching the nose and yawning) caused high numbers of false positives for the artificial neural network. By utilizing the rule-based layer to establish a minimum number of puffs within a smoking session, single gestures such as a yawn will be eliminated as a smoking event. The operational directives of the rule-based core are described later in the paper.

### Training of the Artificial Neural Network

**Figure 3:**
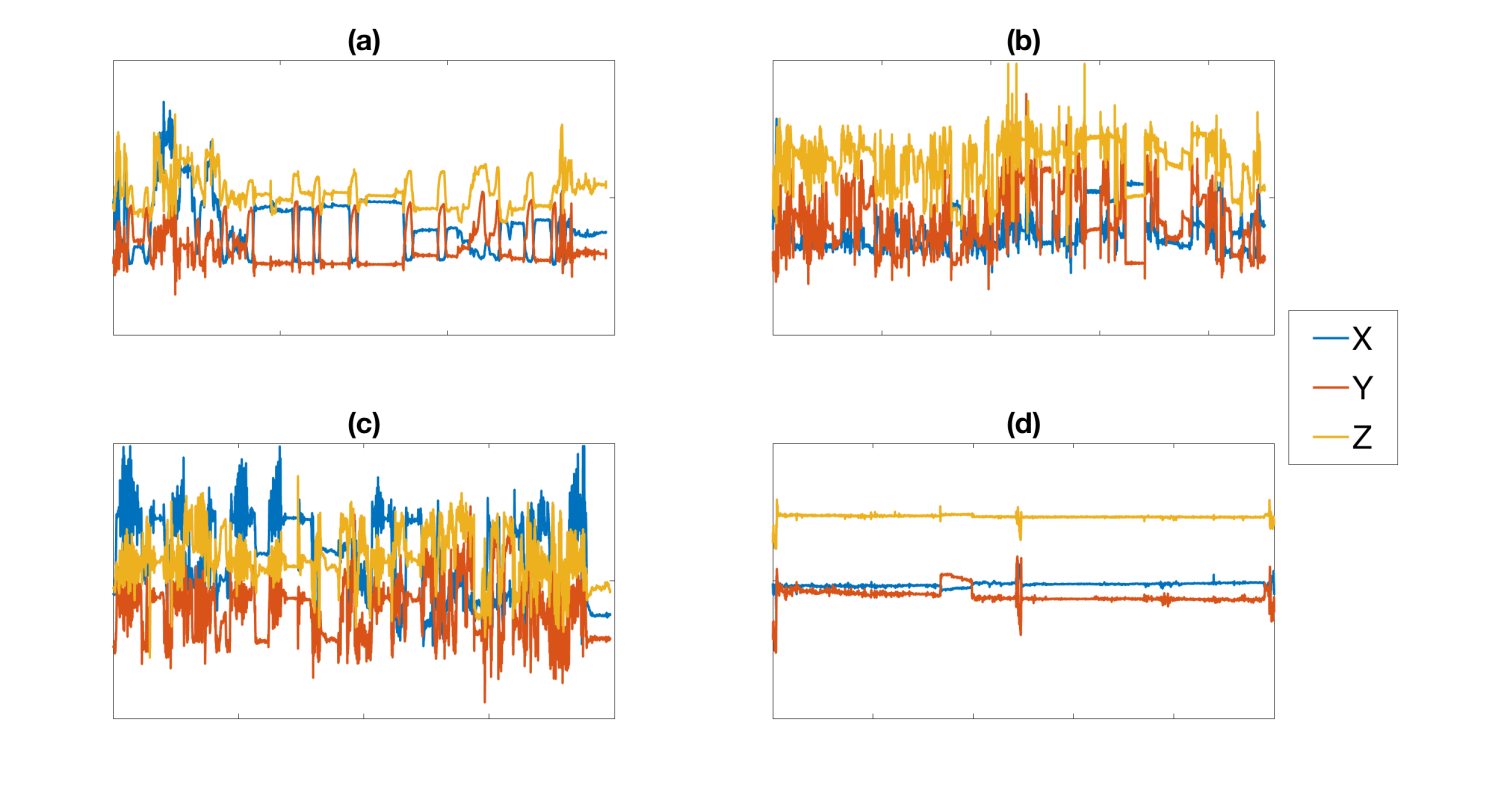
Examples of the following nonsmoking sessions: (a) drinking, (b) eating, (c) walking, and (d) typing on a computer.

It is typical to train the artificial neural networks on a separate set of data than what is used during the validation step to establish its full functionality (to enforce generalization). This process eliminates the possibility of memorization [19] by the AI. Therefore, the training of the artificial neural network was performed with smoking data collected from 10 volunteers that were not part of the data collection mentioned in the “Recruitment of Participants” section. These volunteers were instructed to use the same mobile phone and smartwatch used in the trial to record smoking and nonsmoking sessions in a laboratory setting. The training set consisted of 13 smoking sessions that were collected from 7 of 10 participants and 12 nonsmoking sessions from 3 of the remaining participants. The nonsmoking sessions included a variety of activities such as eating (3 sessions), drinking (3 sessions), walking (3 sessions), tying shoes (1 session), and typing on a computer (2 sessions). An example of each gesture is shown in Figure 3. Inputs to the network were extracted using a 5-second rolling window, which resulted in a total of 177,450 smoking gestures and 174,080 nonsmoking gestures. The smoking gestures were then coded as positive responses and the nonsmoking gestures as negative responses. The artificial neural network was trained and validated with this set of data, achieving an accuracy of 95%. Here we define accuracy to be the percentage of correctly predicted smoking and nonsmoking gestures.

### Development of the Rule-Based Artificial Intelligence From a General Model of a Smoking Session

Precise definition of a smoking session is critical for evaluation of a predicted model and development of any rule-based criteria. Development of a template for a smoking event is beneficial in a number of ways. First, such a definition can be used to compare the output from our detection mechanism to the actual smoking session recorded by participants. Second, the existence of such a model will help to better define the operating rules of the rule-based AI in improving the detection rates.

**Figure 4:**
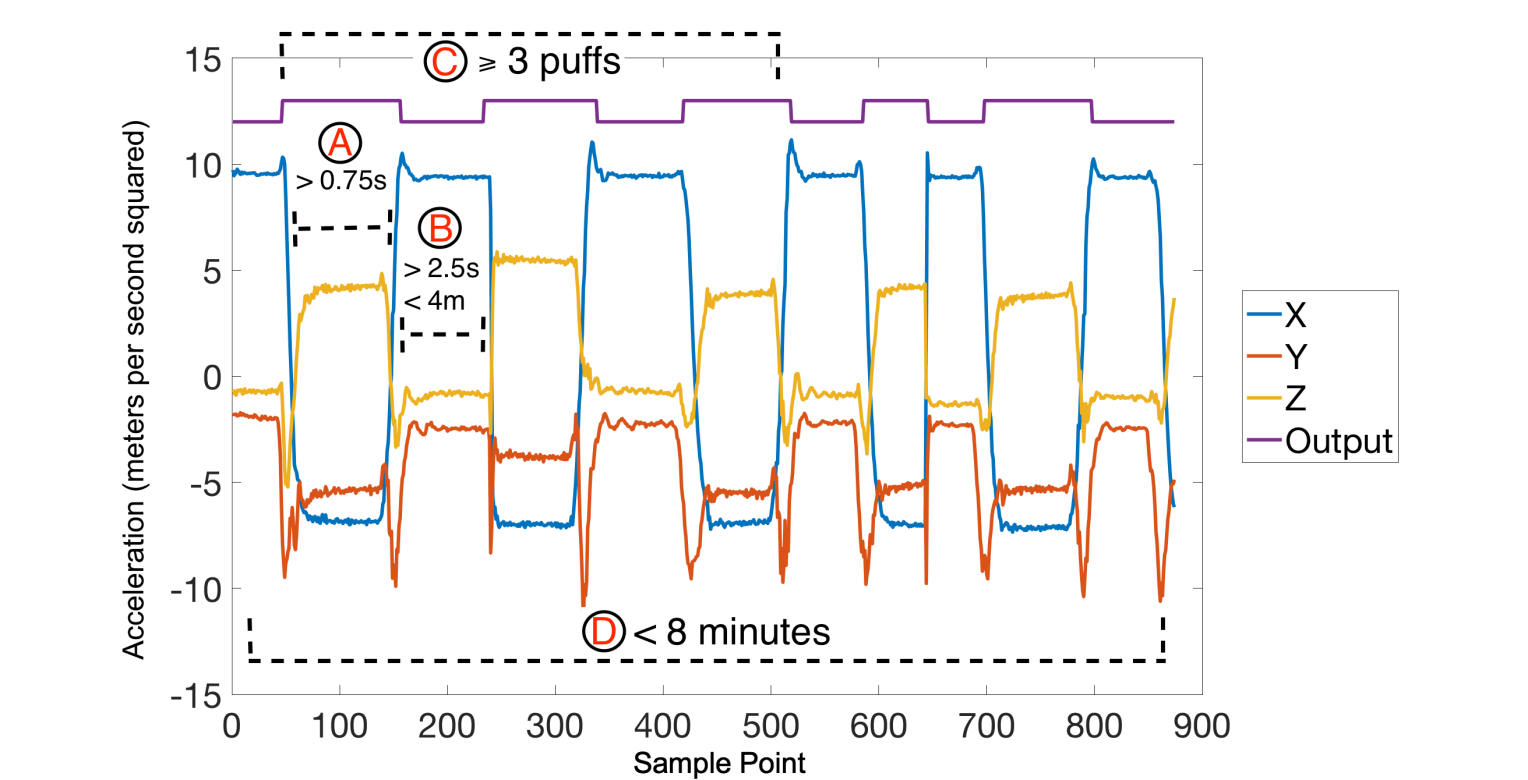
Model of a smoking session: (a) puff duration >0.75 seconds, (b) maximum rest time between puffs <4 minutes and minimum rest time >2.5 seconds, (c) minimum number of puffs in a session=3 puffs, (d) session duration <8 minutes.

A smoking session can be defined in terms of its dependent components such as the number of individual gestures and their time dependencies. Figure 4 describes the model of smoking that was empirically derived based on our observations of the participants’ data. Based on this model, a smoking session is described by five main parameters: minimum puff duration, minimum and maximum rest time between puffs, maximum session duration, and the minimum number of puffs per session. A “puff” was defined as the time it takes a person to raise the cigarette to their lips, inhale, and then lower their arm back to the resting position. Therefore, we conservatively define a minimum puff duration consisting of 0.75 seconds (shown in Figure 4a). Any puff shorter than 0.75 seconds in duration was therefore rejected as a valid puff by the rule-based AI system.

A minimum of 2.5 seconds and a maximum of 4 minutes were used as the rest time that separated two adjacent puffs (Figure 4b) belonging to the same smoking session. Two adjacent puffs in violation of the minimum separation criterion were classified by the rule-based system as the same puff that was incorrectly separated from each other. Correspondingly, two adjacent puffs in violation of the maximum separation criterion are classified to belong to two separate smoking sessions.

Finally, a smoking session was defined to consist of at least 3 puffs that satisfy the previous gesture criteria (eg, puffs must be longer than 0.75 seconds in duration and more than 2.5 seconds and less than 4 minutes from the next puff) and not exceed 8 minutes in duration (Figure 4c-d). The 8-minute rule was implemented to have a higher precedence over all other rules. A sequence of appropriate puffs that exceed 8 minutes in total length was counted as two separate smoking sessions. This rule was primarily implemented to address chain-smoking behavior.

In our data, puff duration never exceeded 5 seconds in length. Therefore, the input to the artificial neural network’s gesture recognition system consisted of a set of accelerometer data that spanned 5 seconds of observation sampled at 20 Hz (100 points of data). Each set of data included x, y, and z components of the accelerometer, which necessitated an artificial neural network architecture with 300 input points and one output point. The single output of the artificial neural network was interpreted based on a threshold of a probability of 0.85, above which signified a smoking gesture. For more details related to the interpretation of the artificial neural network’s output, refer to our previous articles [14,15].

During the supervised training of the artificial neural network, the onset and offset of the smoking gesture was loosely defined by the supervisor. Loose interpretation of the edge is not consequential because it is a very quick event (in comparison to the gesture itself) and therefore makes very little impact on the duration of a gesture.

### Evaluation Techniques

Evaluation of automated methods for detection of smoking gestures can be performed at various levels of granularity. At the finest point, every sampled data point (20 points every second) can serve as the subject of evaluation, whereas at the coarsest point an entire smoking session can be the subject of evaluation.

In this work, we define our objective as successful detection of each smoking session. The interpretation rules of a smoking session (Figure 4) were used to quantify the output of the smoking detection mechanism. The validity of each detected session was established based on comparison to the self-report by the participants. A detected smoking session was categorized as a true positive if it was corroborated by the timestamps of the self-report and false positive otherwise. The true positive rate was measured by the number of detected true positive sessions divided by the total number of smoking sessions reported by the participants.

It is common to provide a measure of false positive rate to form a more complete evaluation of a predictive system’s performance. Calculation of the false positive rate is the total number of nonsmoking sessions that were predicted as smoking divided by the total number of nonsmoking sessions. However, in this instance proper calculation of the total number of nonsmoking sessions term became ambiguous. Within a 12-hour of recording session, a total of 854,400 nonsmoking sessions (of 8 minutes length at 20 Hz of sampling rate) can be extracted via a rolling window. Given that the smoking detection mechanism produced on average two false smoking sessions per participant, the estimated false positive rate would be 2.34×10^-6^. A more meaningful measure of the false positive rate can be achieved by calculating the number of nonsmoking sessions as the total number of contiguous nonsmoking sessions (ie, the number of 8-minute nonsmoking sessions that had no overlap with other ones). The number of nonsmoking sessions was calculated as the total number of minutes recorded by a given participant divided by the window size (in our case 8 minutes) and subtracting the total number of smoking sessions recorded by the participant from this value: number of nonsmoking sessions=(total number of minutes recorded sessions/window size)–total number of smoking sessions. Using this calculation, for a given 12-hour period in which a participant smoked 10 times, the number of smoking sessions would be 80.

## Results

### Summary of Data

#### Participant Demographics

Three of the 10 participants did not complete the demographic survey. Of the participants who completed the survey, the mean age was 32 (SD 6) years, the minimum age was 27 years, and the maximum age was 46 years. There were four females and three males. Six participants were non-Hispanic white, whereas one was African American. Only one participant indicated that they intended to quit smoking within the next 6 months.

#### Participant Data

**Figure 5:**
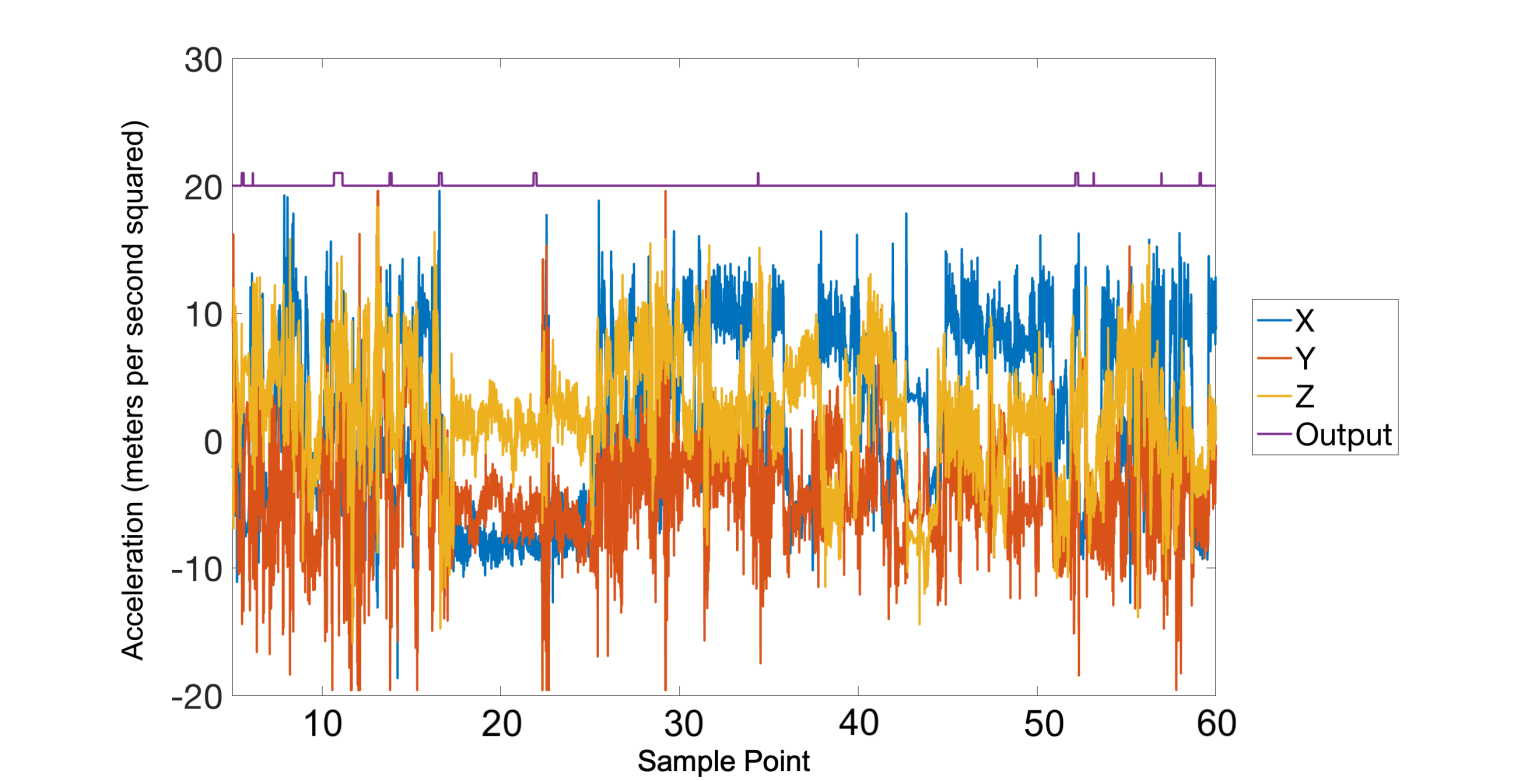
A noisy nonsmoking session is shown a before the smoothing filter with the output of the detection mechanism shown in purple.

**Figure 6:**
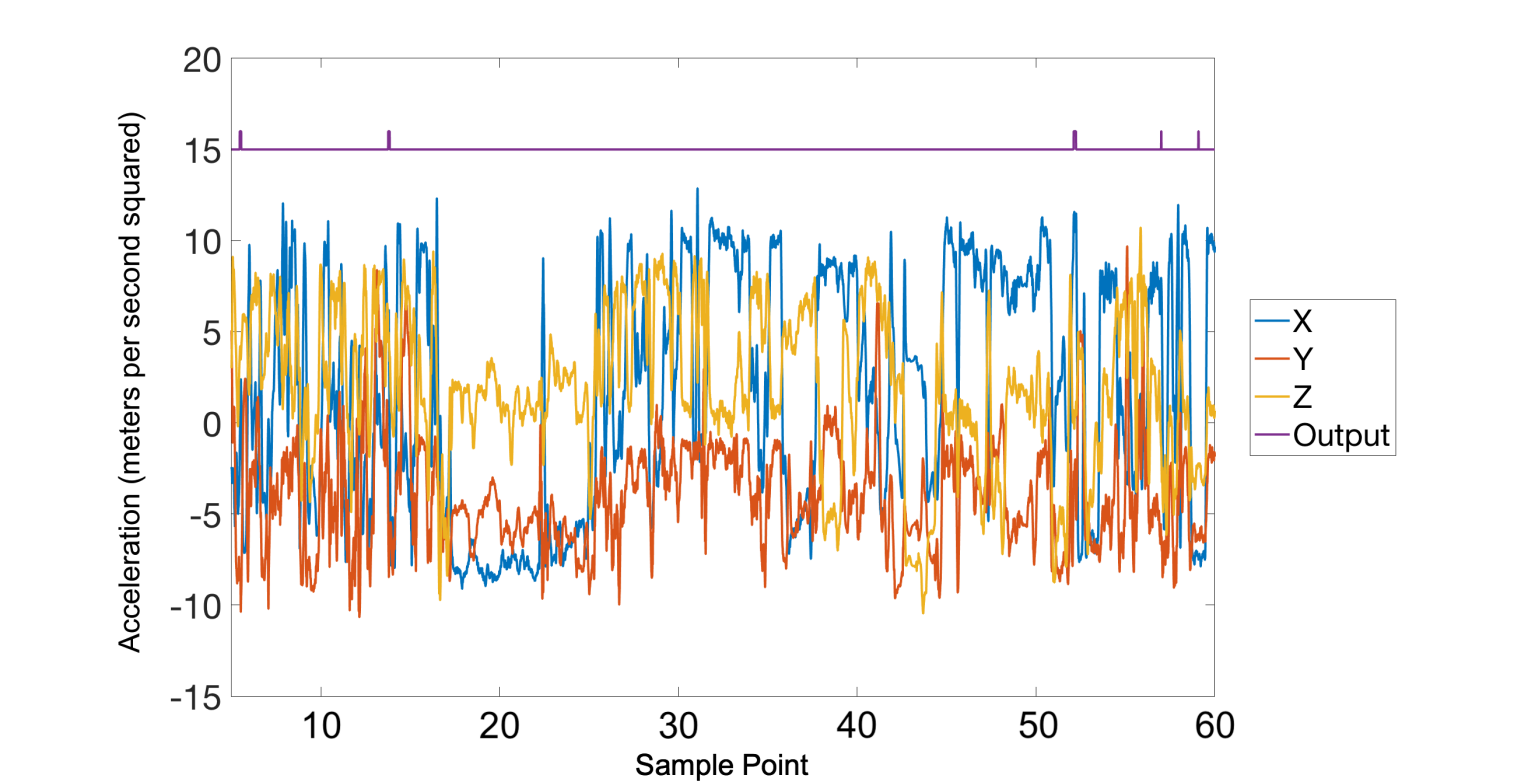
A noisy nonsmoking session is shown after the smoothing filter with the output of the detection mechanism shown in purple.

In total, 120 hours of data were collected from the 10 participants, in which 123 smoking sessions were reported. Each data file was first subjected to a low-pass filter to eliminate the high-frequency noise caused by movements such as walking or shaking. The effect of the filter can be seen in Figures 5 and 6. Following the smoothing step, the inputs to the artificial neural network were prepared by using a rolling window of 5 seconds.

Within the 12 hours of recording, participants typically smoked 12 times. On average, the duration of a smoking session was 8 minutes based on the self-report data and 5 minutes based on visual inspection of the recorded sessions. These discrepancies were most likely a consequence of both the additional time required for manual entry in the self-report protocol and human error. Requiring the participants to log their smoking session in an electronic form may have taken some participants a few extra minutes, thus inflating their reported session window.

### Evaluation Outcomes

#### Self-Reporting Accuracy

**Figure 7:**
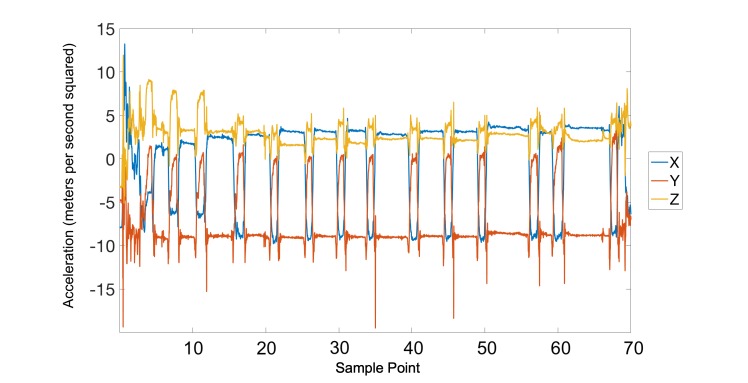
This session was not reported by the participant, but is an unmistakable smoking session with 13 clear puffs.

**Figure 8:**
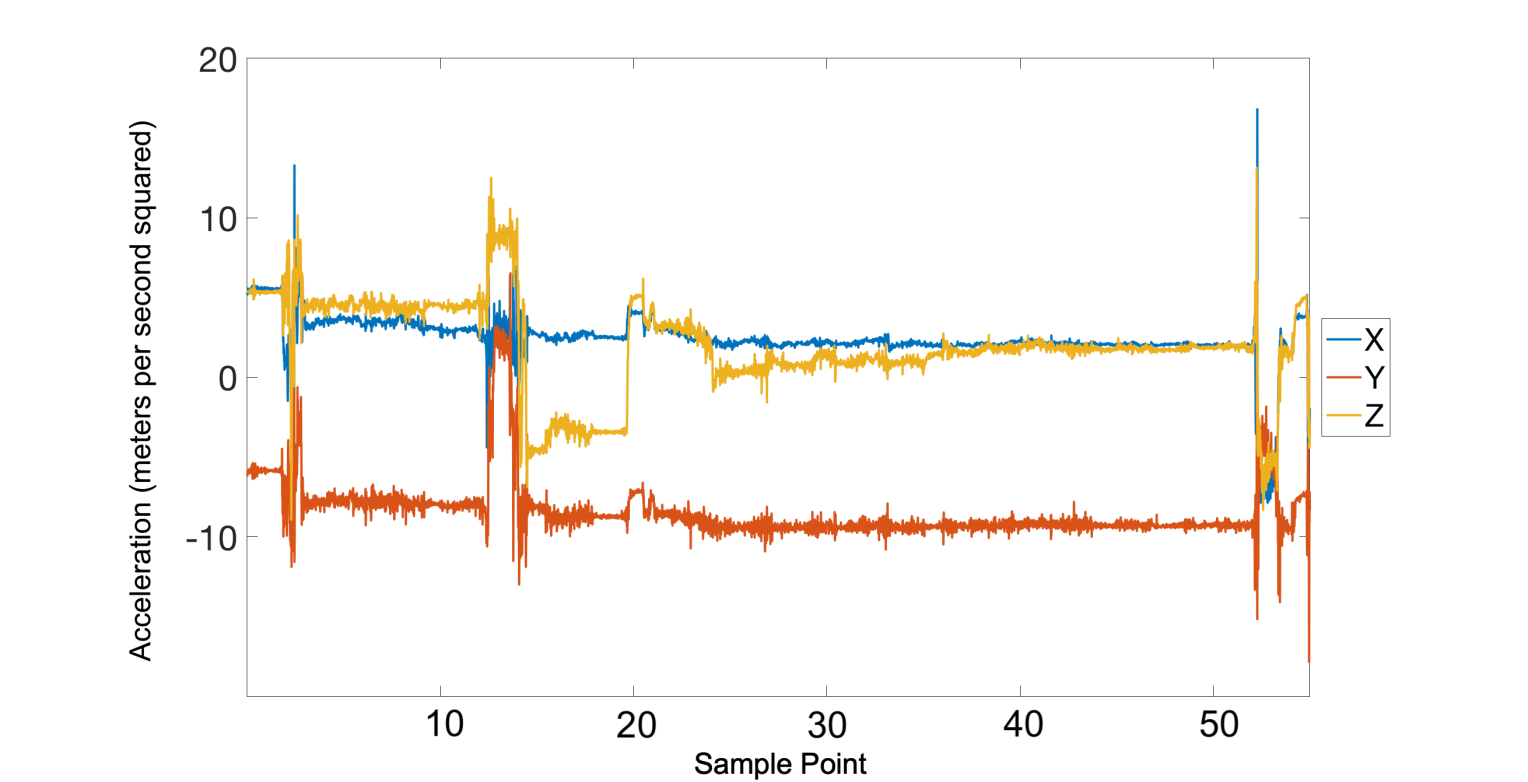
This session was reported as a smoking session, but no clear smoking gestures can be identified.

In total, of the 123 recorded sessions, 27 entries were missing either a start or end time. In these cases, a window of 8 minutes was given preceding an end time with a missing start time or following a start time with a missing end time. Using this metric, the accuracy of self-report (ie, the rate of correctly logged smoking entries) was approximately 78% (96/123). However, it should be noted that we expect the self-report to be lower than the estimated 78%. This expectation is based on close examination of the raw recorded data that would otherwise be impossible to ascertain from self-report data. One such example is shown in Figure 7, where the participant did not report a clear smoking session. However, by a close comparison of the recorded session to the model of smoking, one can make a reasonable determination that it is indeed an unreported smoking event. The omission of this session in the log resulted in an increase of the false positive rate, where it should have contributed to an increase in the true positive rate. The opposite of this phenomenon also occurred; that is, a smoking session was reported in a given period yet on careful inspection no valid smoking event was found in the recorded data (an example is shown in Figure 8). If both of these phenomena were included in the calculation of the self-report accuracy, then it would drop to 71% (88/123 correctly reported sessions).

**Table 1:** Values for the true positive rate calculated by iteratively excluding sessions from the four categories producing false negatives.

Category	Detected smoking sessions, n	Excluded smoking sessions, n	Corrected smoking sessions, n	True positive rate, %
Ground assumption	100	0	123	81
No smoking	100	2	121	82
Improper use	100	9	112	89
Abnormal gesture	100	9-11	101-112	89-99
True false negative	100	0	101-112	89-99

#### Detection Accuracy

The evaluation of the results was not as intuitive as expected. Our initial approach to evaluation (first entry in Table 1) was to compare the outcomes of the automated detection mechanism to that of self-reported smoking. However, this approach presumed 100% accuracy of self-report, for which we have cited some contradictory examples (refer to the previous section). If we assume that self-report is 100% accurate, then real errors in self-report (see previous section) lead to underestimating true positive detection rates and overestimating false positive rates. Therefore, it is paramount to examine and categorize the sources of discrepancy between the two methods. To that end, we define the following categories of discrepancies: “no smoking,” “improper use,” “abnormal gesture,” and “true false negative.” All subsequent investigations of the self-report data were performed by visual inspection of the recorded signals. All detection estimates were adjusted incrementally as each source of error was eliminated. The modified results are shown in Table 1.

The first category of no smoking denotes no visual presence of a smoking event during the reported smoking period (an example is shown in Figure 8). Two such sessions belonged to one participant. These sessions were excluded from the total number of self-reported smoking sessions, which resulted in a new true positive rate of 82% (100/121).

The second category, improper use, was one of the biggest contributors in reducing the true positive rate in this study. Improper use denotes the condition where the participant did not wear the watch as dictated by the protocol of the study (either not on the right wrist or not in the protonated position). This condition can easily be identified and corrected [15], although the correction mechanism was not implemented and incorporated into this study. A total of nine sessions were identified via visual inspection to be in violation of proper adherence to the study protocol and could therefore be excluded from the study. A corrected true positive rate value of 89% (100/112) was estimated after elimination of these violations.

**Table 2:** Values for the false positive rate calculated by iteratively excluding sessions from the two categories producing false positives.

Category	Detected false smoking sessions, n	Excluded sessions, n	Corrected false smoking sessions, n	Total possible sessions, n	False positive rate, %
Ground assumption	22	0	22	777	2.8%
Clearly smoking	22	6	16	771	2.1%
True false positive	22	0	16	771	2.1%

The third category, abnormal gesture, denotes the occurrence of smoking gestures that could not be reproduced in the laboratory setting. These gestures had a clear periodicity consistent with smoking behavior, but had no other resemblance to our database of smoking gestures. Such conditions may be indicative of smoking in unusual positions, such as smoking while lying in the facedown position (possibly from the edge of the bed) or hanging upside down. Various reclined positions, laying down in the face-up position, or lying down on the left or right side were investigated without any success in recreating the recorded anomalous smoking gestures. In future iterations of the detection mechanism used in this study, smoking in these positions should be included in our training session of the artificial neural network. However, before retraining the artificial neural network, these curious gestures need to be confirmed as valid smoking sessions and be reproducible in laboratory settings. Depending on whether such gestures can be excluded from this study or not, an upper bound of 99% accuracy can be estimated for the performance of the automated detection mechanism.

The fourth and final category, true false negative, represented the cases where the self-reporting data were correct, but the automated detection mechanism misidentified the sessions. Our thorough investigation identified only one such session. We suspect the abnormally short puffs by this participant as the culprit for this misclassification. The likelihood of this type of misclassification can be reduced in the future by allowing personalization of the puff duration based on a given person’s smoking profile.

In our evaluation of the false positive rates, we faced the same challenges as in our evaluation of the true positive rate. A progressive evaluation of the false positive rate is shown in Table 2. Under the simplified conditions (assumption of 100% accuracy in self-reporting), a total of 22 smoking sessions were identified within nonsmoking regions of the 120 hours of total recording time. Based on the definition of false positive rate presented in a previous section, 120 hours of recording time translated into 777 windows of observed nonsmoking behavior. Similar to the case of true positive rate, the following categories of false positive rate were investigated to understand the nature of the detection mechanism’s performance better: clearly smoking and true false positive. The results of this classification are summarized in Table 2.

Under the conventional technique of assuming 100% confidence in self-reporting data, on average, the detection mechanism achieved a false positive rate of 2.8% (22/777). However, due to clear presence of errors in self-reports, 2.8% served as an upper bound estimate of performance, and the actual performance can be expected to be lower than 2.8%.

To obtain a better estimate of the false positive rate, the first category of clearly smoking was scrutinized (Table 2). The clearly smoking category denotes sessions in which at least one smoking event was indisputably present, yet no smoking event was logged during the self-reported period of smoking. An example of the phenomenon is shown in Figure 7. A total of six such sessions were identified during a careful manual inspection of the recorded data. It is unclear whether such instances should be included in the evaluation of the true positive rate or false positive rate. Here we have chosen the latter and have excluded them from the calculation of the false positive rate. With these excluded, the false positive rate was reduced to 2.1% (16/771).

The second category, true false positive, signified the cases where the smoking detection mechanism performed a true misclassification and thus could not be excluded. A total of 16 such sessions fell into this category. The majority of these sessions contained very jittery and erratic motions, which may be the cause of their misclassification. If so, a more rigorous filtration of high-frequency signals may remove or reduce this category of error in future iterations of the software.

## Discussion

### Principal Results

The presented automated smoking detection mechanism demonstrated a conservative true positive rate of more than 82% for identifying smoking sessions, while achieving a negligible false positive rate of 3%. Furthermore, the true positive rate increased to approximately 90% when considering only the smoking sessions that participants adhered to study protocols. Approximately 10 of the smoking sessions were not reproducible in the laboratory session, which will be the subject of future studies to assess how different smoking positions (eg, while lying down) are accompanied by different gesture patterns or otherwise influence accelerometer readings. Once confirmed as valid smoking sessions, similar gesture patterns can be included in future training sessions of the detection mechanism’s underlying artificial neural network. A new true positive rate can be estimated for the newly trained artificial neural network by assuming 50% successful detection of the anomalous gestures (although, based on the current true positive rate, 80% is more realistic). A 50% success rate in detecting anomalous gestures will increase the true positive rate to 93% accuracy. In contrast, a liberal assessment of the traditional self-report had a maximum accuracy of 71% to 78%. However, we speculate actual accuracy of self-report may be lower if our analysis of the data from our study is indicative of normal self-report behavior.

### Limitations

There are two primary limitations of the automated, machine learning-based approach to detection of smoking: technological and methodological. Technological aspects include the battery life span, which is of primary interest for apps that require continuous monitoring over waking hours. The wearable device used in our studies (Zen watch) has a limited practical battery life of nearly 20 hours. However, this battery life span may be significantly reduced under high-throughput data exchange conditions, where data are continuously transmitted to another device via a Bluetooth connection. Although a limitation for practical deployment of an automated smoking detection approach, limited battery life can be mitigated in two ways. First, the identification of puffs, smoking gestures, and smoking sessions can be translocated on the watch and therefore eliminate excessive Bluetooth communication. We anticipate a substantial reduction in the power consumption of the smartwatch, returning its life span to nearly 10 hours a day. The second mitigation of limited battery life is newly arriving smartwatches with battery life spans of more than a week. Therefore, the prospect of continuously monitoring smoking behavior for a day or more is highly positive.

A number of methodological issues also limited this study. The first issue is related to study protocol adherence, which requires participants to wear the smartwatches in a particular fashion (eg, wearing the watch on the dominant hand). Although these protocols may be acceptable during the early stages of a study, they may be cumbersome during the broader dissemination of this approach. To that end, our existing algorithm should be improved to detect the orientation of the smartwatch (left hand versus right hand, supinated or pronated) either automatically or during the initial setup stages. Our subsequent work [15] has demonstrated the possibility for automatic correction of the accelerometer data if the watch is worn incorrectly. In addition, study procedures can warn the user if the watch is not worn correctly. The second issue is related to the anomalous and irreproducible smoking gestures we observed. These gestures need to be further studied and, once confirmed as valid smoking gestures, examples need to be included in future iterations of the smoking detection mechanism.

### Conclusions

The potential benefits of developing an automated system for detection of human activities are vast. Based on our observations, two distinct conclusions can be stated. First, it is possible to detect smoking behavior based on triaxial accelerometer data and this behavior can be distinguished from other similar gestures. Second, an automated smoking detection approach to the study of smoking behavior may be substantially more reliable than approaches that rely on traditional self-report. Third, with an accurate, automated system in place, reliance on self-reporting could be eliminated, thus decreasing the burden on a participant without losing any benefits. The resulting data collection system could allow for a range of unobtrusive studies of how context, including that which can be captured by global positioning systems, influences smoking behavior, targeted surveys around smoking events, and targeted communications for those who are trying to quit. Furthermore, this automated system may easily be expanded to detect increasingly popular electronic cigarette smoking, for which behavioral gestures accompanying consumption are very similar to cigarette smoking but for which the patterns of behavior and their context are much less well understood.

## Abbreviations

AI: artificial intelligence
